# Heading Frequency Is More Strongly Related to Cognitive Performance Than Unintentional Head Impacts in Amateur Soccer Players

**DOI:** 10.3389/fneur.2018.00240

**Published:** 2018-04-24

**Authors:** Walter F. Stewart, Namhee Kim, Chloe Ifrah, Martin Sliwinski, Molly E. Zimmerman, Mimi Kim, Richard B. Lipton, Michael L. Lipton

**Affiliations:** ^1^Sutter Health Research, Walnut Creek, CA, United States; ^2^The Gruss Magnetic Resonance Research Center, Bronx, NY, United States; ^3^Department of Radiology, Albert Einstein College of Medicine, Montefiore Medical Center, Bronx, NY, United States; ^4^Department of Human Development and Family Studies, Pennsylvania State University, University Park, PA, United States; ^5^Department of Neurology, Albert Einstein College of Medicine, Montefiore Medical Center, Bronx, NY, United States; ^6^Fordham University, Bronx, NY, United States; ^7^Department of Epidemiology and Population Health, Albert Einstein College of Medicine, Montefiore Medical Center, Bronx, NY, United States; ^8^Department of Psychiatry and Behavioral Sciences, Albert Einstein College of Medicine, Montefiore Medical Center, Bronx, NY, United States; ^9^The Dominick P. Purpura Department of Neuroscience, Albert Einstein College of Medicine, Montefiore Medical Center, Bronx, NY, United States

**Keywords:** risk factors in epidemiology, brain trauma, heading, sports, soccer

## Abstract

**Objective:**

Compared to heading, unintentional head impacts (e.g., elbow to head, head to head, head to goalpost) in soccer are more strongly related to risk of moderate to very severe Central Nervous System (CNS) symptoms. But, most head impacts associated with CNS symptoms that occur in soccer are mild and are more strongly related to heading. We tested for a differential relation of heading and unintentional head impacts with neuropsychological (NP) test performance.

**Method:**

Active adult amateur soccer players were recruited in New York City and the surrounding areas for this repeated measures longitudinal study of individuals who were enrolled if they had 5+ years of soccer play and were active playing soccer 6+ months/year. All participants completed a baseline validated questionnaire (“HeadCount-2w”), reporting 2-week recall of soccer activity, heading and unintentional head impacts. In addition, participants also completed NP tests of verbal learning, verbal memory, psychomotor speed, attention, and working memory. Most participants also completed one or more identical follow-up protocols (i.e., HeadCount-2w and NP tests) at 3- to 6-month intervals over a 2-year period. Repeated measures General Estimating Equations (GEE) linear models were used to determine if variation in NP tests at each visit was related to variation in either heading or unintentional head impacts in the 2-week period before testing.

**Results:**

308 players (78% male) completed 741 HeadCount-2w. Mean (median) heading/2-weeks was 50 (17) for men and 26 (7) for women. Heading was significantly associated with poorer performance on psychomotor speed (*p* < 0.001) and attention (*p* = 0.02) tasks and was borderline significant with poorer performance on the working memory (*p* = 0.06) task. Unintentional head impacts were not significantly associated with any NP test. Results did not differ after excluding 22 HeadCount-2w with reported concussive or borderline concussive symptoms.

**Conclusion:**

Poorer NP test performance was consistently related to frequent heading during soccer practice and competition in the 2 weeks before testing. In contrast, unintentional head impacts incurred during soccer were not related to cognitive performance.

## Introduction

More than 1 in 20 humans currently play soccer, the only sport where athletes deliberately engage in repeated head impacts (1, 2). Growing evidence indicates that soccer is associated with risk of persistent central nervous system (CNS) impairment and structural changes (3–10). These long-term effects may be mediated by short-term effects of soccer (9–12).

Most studies of head injury in other sports have focused on single or repeated recognized concussions that are associated with risk of cognitive impairment (3, 13, 14) and chronic brain pathology (15), especially in susceptible individuals (16, 17). Years of soccer play are associated with neuropsychological (NP) impairment (8–23), abnormal white matter microstructure (8, 24), and structural brain changes (25, 26). Impairments to memory (17–24), attention (8, 9, 22, 26), and cognitive flexibility (9, 19, 23) are the domains of cognitive function most commonly associated with soccer play. However, most previous studies have focused on the long-term effects of heading or head injury. Fewer studies have examined the short-term effects of soccer activity on CNS function (5, 10, 27).

Variation in CNS symptoms and cognitive function are associated with soccer activity (10–12, 27–30). While concussions and unintentional head impacts have been a focus of concern for significant sequelae from soccer (25, 31), heading may also be an important mediator. Our prior work indicates that intentional heading is far more frequent than unintentional head impacts (e.g., head to head, head to goalpost), but heading impacts are less strongly associated with CNS symptoms (10). While recent evidence indicates that heading is directly associated with short-term impairment to cognitive function, no study to date has quantified the differential impact of heading versus unintentional head impacts on variation in neurobehavioral function.

Our prior work indicates that CNS symptoms related to head impacts in soccer are common (28). Specifically, we found that among active adult amateur players, CNS symptoms were reported to occur in 74% of 2-week recall periods (28) and that the 2-week recall of unintentional head impacts was more strongly related to moderate to very severe CNS symptoms than was heading (28), a finding that is consistent with reported causes of concussion in soccer (1, 28). But, 73% of reported symptoms were mild and heading accounted for a majority of all symptoms. While concussion risk in soccer is currently the dominant concern in the medical literature and lay press, repetitive heading may contribute to accumulated changes that influence long-term risk, even though its acute manifestations are more subtle.

In this study, we examined whether self-reported (11, 28, 32, 33) unintentional head impacts or heading in a 2-week period explained variation in performance on a battery of NP tests.

## Materials and Methods

The Einstein Soccer Study is a multi-faceted longitudinal study of heading and its consequences in adult amateur soccer players. We used data from one sub-study, where soccer players were recruited over a 37-month period between November 2013 and January 2017 and to complete one to seven [max number of sessions] sessions where a battery of NP tests was completed along with a 2-week recall questionnaire (“HeadCount-2w”) on soccer activity, heading, and other head impacts. Sessions were completed every 3 to 6 months for up to [max number of sessions]. Head impact and NP testing data collected during a single session was the unit of analysis, where each soccer player could contribute one or more units. In this study, we used data across 741 visits from 308 soccer players to determine if variation in NP test performance was explained by variation in either heading or unintentional head impacts reported for the preceding 2 weeks. The Albert Einstein College of Medicine IRB approved the study. All participants gave written informed consent at time of enrollment.

### 2-Week Recall Questionnaire

Details on the development of HeadCount-2w, the 2-week recall questionnaire, are described elsewhere (28). In brief, HeadCount-2w was completed as a web-based questionnaire, organized into four modules focused on heading in outdoor practice, outdoor games, indoor practice, and indoor games. Identical questions are repeated for outdoor and indoor settings and include: (1) any practice sessions; (2) number of soccer practice days; (3) average number of headings during practice in the past two- weeks; and (4) CNS symptoms. Participants were asked how often (0 to 5+ episodes) in the past 2 weeks they experienced heading that was directly followed by CNS symptoms, defined as very low impact (no pain = 0), mild impact (slight pain = 1), moderate impact (moderate pain, some dizziness = 2), severe impact (felt dazed, stopped play, needed medical attention = 3), and very severe impact (knocked unconscious = 4). For indoor and outdoor competition, questions were asked about the: (1) number of competitive soccer games; (2) positions played during games; (3) average number of headings during games; and (4) CNS symptoms (as above). In addition to these sections on heading, participants were asked, for all soccer activities in the past 2 weeks, how often they experienced unintentional head impacts, such as a player’s head hitting another player’s head, elbow, knee, or the ground, or being hit by the ball in the back of the head, among others. Finally, questions were asked about lifetime head injuries. HeadCount-2w has been previously validated (32, 33).

### NP Testing

The NP assessment was completed during an in-person session within the 2-week recall period of the web-based HeadCount-2w questionnaire. Cogstate^®^ (Cogstate, Ltd., NY, USA), a valid and reliable computer-administered battery of cognitive function (34) was administered. Tests were selected to assess five NP domains. Performance metrics from each task were employed as dependent variables. The International Shopping List Task, immediate and delayed recall (ISL, ISRL), measured verbal learning and memory abilities by asking participants to remember a list of words on three consecutive learning trials and then to recall the list following a 20-min delay. Number of correct responses was used in the analysis. Psychomotor speed was assessed with the Groton Maze Chase Task (GMCT), which measured how quickly and accurately (total number of correct moves per second) participants chased a target through a maze. The Identification task (IDN) assessed attention by measuring how quickly (log_10_ of reaction time) participants correctly identified the color of a playing card. The One Back task (ONB) also assessed attention by measuring how accurately (arcsine of square root of proportion of correct responses) participants determined if a playing card was the same as the card shown previously. Working memory was assessed with the Two Back task (TWOB), which measured how accurately (arcsine of square root of proportion of correct responses) participants determined if a playing card was the same as the card shown two cards previously.

### Study Population and Data Collection

Details on the study and recruitment methods are described elsewhere (28). In brief, adult amateur soccer players, recruited through a diversity of outreach methods in New York City and surrounding areas, were directed to an enrollment website for preliminary consent and screening. Inclusion criteria were: age 18–55; at least 5 years of active amateur soccer play; current active amateur soccer play; 6+ months of amateur soccer play per year; and English language fluency. Exclusion criteria were: schizophrenia; bipolar disorder; neurological disorder; and pregnancy.

Written informed consent occurred during an initial in-person study visit during which Cogstate^®^ was administered by a trained research assistant. At the end of the study visit, each subject was told they would receive an email message asking them to complete an online HeadCount-2w survey to report 2-week recall of soccer activity. Compensation for the study visit ($150.00) was contingent on completion of the online HeadCount-2w within a week of the study visit. Subjects were similarly asked to complete HeadCount-2w questionnaires and NP testing in conjunction with subsequent study visits that occurred every 3–6 months (depending on study arm) after the baseline visit. Of 994 HeadCount-2w completed, 226 were excluded from the analysis because no soccer activity was reported.

Of the remaining 768 HeadCount-2w, 7 were excluded because heading data reported by one female were extreme outliers (i.e., 364 to 795 headings/2 weeks) and another 20 were excluded because NP data were not collected because the in-person visit was not completed. A total of 308 individuals participated in 741 sessions where the HeadCount-2w and NP test battery were completed. A total of 129 individuals contributed data for one session, 70 contributed data from two sessions, and 112 individuals contributed data from three or more sessions. Of the 741 HeadCount-2w with NP data, 36 were missing the ONB because this task was added to the Cogstate^®^ Battery after study inception. Six administrations of the GMCT and 10 of the IDN were excluded due to technical problems that caused data loss.

### Analysis

Data used for this analysis was from the 741 sessions (i.e., visits) where both NP tests and HeadCount-2w were completed by 308 soccer players. Analysis determined if variation in scores on specific NP tasks completed at each visit was explained by variation in heading or of unintentional head impacts reported to have occurred in the 2-weeks preceding NP testing.

Total heading was highly skewed and, therefore, defined as an ordinal-categorical variable of approximately equal size quartiles with the lowest exposure group having de minimis heading exposure (i.e., 0 to 4 headings in 2 weeks). Unintentional head impacts were represented as an ordinal variable (i.e., 0, 1, 2+ events).

Generalized estimating equations (GEE) repeated measures linear regression was used to determine the relation of NP test scores with heading (i.e., four ordered categories) and unintentional head impact (i.e., 0, 1, 2+) categories, using the lowest exposure group for each variable as the reference for estimating differences. Covariates considered in the model as potential confounders included performance on the reading subtest of the Wide Range Achievement Test 4 (35) (included in all models), lifetime concussion count, years of soccer play at a similar frequency, sex, age, field position, alcohol use, and cigarette use. Covariates were retained in each model if they exhibited at least a borderline significant (*p* < 0.10) relation to the outcome. Models were first completed with all 741 HeadCounts-2w and then without 14 HeadCounts-2w on which probable concussive symptoms (felt dazed, stopped play, required medical attention) were reported and another 8 HeadCounts-2w on which concussion was reported. The Wald chi-square test was used to determine if either the heading or unintentional head impact variables were related to NP test scores. We used the GEE Model procedure in SPSS Version 24.0.

## Results

Of 741 HeadCount-2w, 246 reported heading activity and unintentional impacts, 435 reported heading but no unintentional impacts, 11 reported unintentional impacts but no heading, and 49 reported no unintentional head impacts and no heading.

### Heading Activity

Among all eligible HeadCount-2w (Table 1, right hand columns), 70.2% reported outdoor competition, followed by 47.9% for outdoor practice, 35.1% for indoor competition, and 18.8% for indoor practice. Notably, the number of days of activity and the headings per day of activity was greater for practice than it was for competition (Table 1). The total mean (median) heading across all activities in a 2-week period was 44.9 (15) for all HeadCount-2w and it was 48.9 (15) for the 681 HeadCount-2w with any heading. Significant right skewness of the heading data is indicated by the striking difference between the mean and median statistics (Table 2). Greater heading was significantly associated with male gender (*p* < 0.01), younger age (*p* < 0.01), soccer position (*p* = 0.05), no alcohol use (*p* < 0.01), no history of concussion (*p* < 0.01), and lower WRAT score (*p* = 0.01). Substantially higher levels of heading were observed for males, younger players, and those with lower WRAT scores. However, after adjusting for all covariates in the same model, heading was significantly associated with the same covariates except for concussion (*p* = 0.07) and WRAT (*p* = 0.12). The latter suggests that the relation of the WRAT score with heading is explained by other confounders.

**Table 1:** Summary of heading and unintentional head impacts reported by 308^a^ amateur soccer players who reported soccer activity in the baseline 2-week HeadCount-2w questionnaire and for all 741 HeadCount-2w questionnaires.

Variable	Baseline HeadCount-2w Questionnaire (*N* = 308)		All HeadCount-2w Questionnaires (*N* = 741)	
**OUTDOOR COMPETITION HEADING**				
Percent with 1+ outdoor game	68.5% (*N* = 211)		70.2% (*N* = 520)	
	Mean	Median	Mean	Median
Number of outdoor games	3.1	2	3.2	3
Headings per game	5.5	4	4.9	4
Cumulative heading in 2-weeks^b^	18.5	10	17.4	10
**OUTDOOR PRACTICE HEADING**				
Percent with 1+ day of outdoor practice	55.2% (*N* = 170)		47.9% (*N* = 355)	
	Mean	Median	Mean	Median
Number of outdoor practices	4.0	3	4.0	3
Headings per practice	8.8	5	7.6	4
Cumulative heading in 2-weeks^c^	38.0	16	48.0	20
**INDOOR COMPETITION HEADING**				
Percent with 1+ indoor game	31.8% (*N* = 98)		35.1% (*N* = 260)	
	Mean	Median	Mean	Median
Number of indoor games	2.7	2	2.8	2
Headings per game	2.8	2	3.1	2
Cumulative heading in 2-weeks^d^	7.5	4	10.3	4
**INDOOR PRACTICE HEADING**				
Percent with 1+ day of indoor practice	19.5% (*N* = 60)		18.8% (*N* = 139)	
	Mean	Median	Mean	Median
Number of indoor practices	2.6	2	2.9	2
Headings per practice	6.2	4	5.7	3
Cumulative heading in 2-weeks^e^	19.7	7	29.1	8
**TOTAL ACTIVITY AND HEADING**				
Any soccer activity	*N* = 308 (274 with heading)		*N* = 741 (681with heading)	
	Mean	Median	Mean	Median
Total heading, all activities, all subjects	39.9	15	44.9	15
Total heading, all activities, heading only	44.9	19	48.9	17
**UNINTENTIONAL EXPOSURE TO HEAD INJURY FROM SOCCER**				
Cause of exposure	Percent exposed		Percent exposed	
Hit in back of head	12.7		13.0	
Head hit goalpost	2.3		1.2	
Head to head	12.3		11.4	
Head hit ground	11.0		9.2	
Head hit elbow, knee	19.8		17.9	
Head kicked	2.6		2.3	
At least one event	36.0		34.7	

*^a^Excludes one soccer player with extreme outlier values for heading activity*.

*^b^*Outdoor competition*: [outdoor competition games in 2 weeks] × [average headings/game]*.

*^c^*Outdoor practice*: [outdoor practice days in 2 weeks] × [outdoor average headings/practice]*.

*^d^*Indoor competition*: [indoor competition games in 2 weeks] × [average headings/game]*.

*^e^*Indoor practice*: [indoor practice days in 2 weeks] × [indoor average headings/practice]*.

**Table 2:** Descriptive statistics on intentional (heading) and unintentional head impacts and neuropsychological (NP) scores by demographic and other factors using data from the 741 HeadCounts-2w and the in-person NP assessments.

**Variable**	**Category**	**Percent^a^ (N = 741)**	**Heading/2-weeks**		**Unintentional head impacts %**	**Mean scores**					
					
			**Mean**	**Median**		**International shopping list**	**Groton maze chase task**	**Identification task**	**One back task**	**Two back task**	**International shopping list recall**
Total		100	44.9	15	36.5	26.7	1.5	2.7	1.3	1.2	9.6

Gender	Male	77.7	50.2^b,c^	17	34.0	26.2	1.6	2.7	1.3	1.2	9.4
	Female	22.3	26.2^b,c^	7	37.0	28.5	1.6	2.7	1.3	1.3	10.4

Age	18–20	23.8	63.5^b,c^	24.5	40.9	26.6	1.6	2.7	1.3	1.2	9.4
	21–23	25.1	58.2^b,c^	24	38.2	26.4	1.6	2.7	1.3	1.3	9.5
	24–28	24.7	27.2^b,c^	10	27.3	27.4	1.6	2.7	1.3	1.20	10
	29+	26.5	32.2^b,c^	13	32.7	26.6	1.3	2.7	1.3	1.2	9.5

Years Playing Soccer at similar frequency	0–8.9	33.6	40.7	12	34.9	27.2	1.6	2.7	1.3	1.2	9.8
	9–14.9	32.7	58.3	21.5	39.3	26.5	1.6	2.7	1.3	1.2	9.4
	15–26.9	26.0	38.5	15	29.5	26.6	1.6	2.7	1.3	1.2	9.7
	27+	7.7	27.9	11	31.6	26.3	1.3	2.8	1.3	1.2	9.3

Position played most often	Forward	19.8	56.1^b,c^	22	45.2	25.8	1.5	2.7	1.3	1.2	9.2
	Midfield	37.3	39.9^b,c^	15	32.7	26.5	1.6	2.7	1.3	1.2	9.4
	Defense	36	48.6^b,c^	15	29.3	27.4	1.5	2.7	1.3	1.2	9.9
	Goaltender	6.9	22.9^b,c^	6	45.1	26.9	1.6	2.7	1.3	1.2	9.7

Concussions (all causes): lifetime	None	79.8	50.4^b^	16	34.2	26.5	1.6	2.7	1.3	1.2	9.5
	1–2	16.6	25.8^b^	12	35.8	27.1	1.5	2.7	1.3	1.2	9.7
	3–4	2.0	15.1^b^	12	33.3	29.1	1.6	2.7	1.4	1.4	10.9
	5–6+	1.6	5.4^b^	3.5	50	29.3	1.2	2.7	1.5	1.3	10.3

Cigarette smoking	Never	71.3	49	16	35	26.3	1.5	2.7	1.3	1.2	9.4
	1+ pack/day	28.7	34.8	13	33.8	27.7	1.5	2.7	1.3	1.3	10.1

Drinks of Alcohol Per Week	Never	29.8	80.3^b,c^	36	40.7	25.9	1.5	2.7	1.2	1.2	9.1
	1–2	35.8	33.7^b,c^	15	32.5	26.7	1.5	2.7	1.3	1.2	9.7
	3–7	26.5	24.1^b,c^	10	32.1	27.4	1.6	2.7	1.3	1.3	9.8
	8+	7.6	32.3^b,c^	11.5	32.1	27.8	1.5	2.7	1.3	1.3	10.2

WRAT Scores	0–96.9	23.2	71.6^b^	31	37.2	24.9	1.5	2.7	1.2	1.2	8.9
	97–104.9	25.6	41.4^b^	16.5	34.7	26.8	1.6	2.7	1.3	1.2	9.6
	105–113.9	26	41.7^b^	15	31.6	26.8	1.6	2.7	1.3	1.2	9.6
	114+	25.1	27.1^b^	9	35.5	28.3	1.6	2.7	1.3	1.3	10.2

*^a^Percent of total HeadCounts-2w by covariates, not individual soccer players*.

*^b^Significant difference (*p* < 0.05) in mean heading by category when tested alone*.

*^c^Significant difference (*p* < 0.05) in mean heading by category after adjusting for other covariates*.

### Unintentional Head Impacts

Among all eligible HeadCount-2w, 34.7% reported one or more unintentional head impacts (Table 1); 15.8% reported two or more. “Head hit elbow or knee” and “ball hit the back of the head” were the two most common types of unintentional impact. Unintentional head impacts were not significantly associated with any of the covariates summarized in Table 2.

### NP Performance and Head Impacts

The median value for heading/2-weeks was 2 for the reference group quartile and it was 9, 25, and 101 for the second through fourth quartiles, respectively. Pearson correlation of the WRAT score with NP tests was 0.05 for the GMCT, −0.09 for the IDN, and between 0.18 and 0.26 for the other four tests.

Using data from the 741 HeadCount-2w and each NP test, separate GEE models were first run with heading or unintentional head impacts, respectively, as the independent variable (Table 3, Column A). Heading was significantly associated with poorer performance on the GMCT (Wald chi-square, *p* < 0.001), One Back (*p* < 0.01), and Two Back (*p* = 0.03). Coefficients for 1 or 2+ unintentional head impacts were consistently negative, but none were significantly related to performance on any of the NP tests (Table 3, Column A). Associations were unchanged after simultaneously adjusting for heading and unintentional head impacts (Table 3, Column B), except that the association of heading with the TWOB (*p* = 0.06) was borderline significant. In addition, coefficients for 1 and 2+ unintentional head impacts were consistently less negative. There were no substantial changes to the associations of heading and NP tests after excluding 22 HeadCounts-2w that reported probable or certain concussive events (Table 3, Column C).

**Table 3:** GEE model estimating the mean difference in NP test scores by heading exposure quartiles and by number of unintentional head impacts reported in a 2-week recall period.

Exposure	Median value	Mean difference in Neuropsychological Test Score							
		
		Includes all 2-week episodes					Excludes episodes with reported probable or certain concussion		
			
		*N*^a^	A^a^ mean difference (95% CI)	Wald chi-square (*p*-value)	B^b^ mean difference (95% CI)	Wald chi-square (*p*-value)	*N*^a^	C^B^ mean difference (95% CI)	Wald chi-square (*p*-value)
			
		*N* = 741	Verbal learning: higher score = better performance^c^ International shopping list—immediate recall (ISL)				*N* = 719		
**Heading**	2	177	–	4.20 (0.24)	–	3.11 (0.37)	173	–	3.49 (0.32)
	9	191	0.34 (−0.40, 1.07)		0.35 (−0.39, 1.10)		186	0.36 (−0.39, 1.10)
	25	187	−0.29 (−1.15, 0.57)		−0.22 (−1.10, 0.67)		181	−0.27 (−1.16, 0.63)	
	101	186	−0.54 (−1.49, 0.42)		−0.40 (−1.44, 0.64)		179	−0.43 (−1.47, 0.61)	

**Unintentional head impacts**	0	484	–	2.38 (0.31)	–	1.30 (0.52)	472	–	1.50 (0.47)
	1	140	−0.08 (−0.82, 0.65)		−0.02 (−0.79, 0.75)		135	−0.05 (−0.83, 0.74)
	2 +	117	−0.73 (−1.67, 0.21)		−0.56 (−1.58, 0.46)		112	−0.62 (−1.65, 0.42)	

		*****N*** = 735**	**Psychomotor speed: higher score = better performance^d^** **Groton Maze Chase Task**				*****N*** = 713**		

**Heading**	2	174	–	24.50	–	22.90	170	–	23.30
	9	191	0.07 (0.00, 0.15)	(0.00)	0.08 (0.01, 0.15)	(0.00)	186	0.08 (0.01, 0.16)	(0.00)
	25	186	−0.01 (−0.09, 0.08)		−0.00 (−0.09, 0.10)		180	−0.01 (−0.08, 0.10)	
	101	184	−0.12 (−0.21, −0.03)		−0.11 (−0.20, −0.01)		177	−0.10 (−0.20, −0.00)	

**Unintentional head impacts**	0	480	–	4.08 (0.13)	–	1.77 (0.41)	468	–	1.85
	1	138	−0.07 (−0.15, 0.01)		−0.05 (−0.12, 0.03)		133	−0.06 (−0.13, 0.02)	(0.40)
	2+	117	−0.08 (−0.18, 0.02)		−0.05 (−0.15, 0.06)		112	−0.03 (−0.13, 0.08)	

		***N* = 732**	**Attention: lower score = better performance^e^** **Identification task (IDN)**				***N* = 711**		

**Heading**	2	176	–	6.00 (0.11)	–	2.54 (0.47)	172	–	3.29
	9	189	−0.01 (−0.02, 0.01)		−0.00 (−0.02, 0.02)		184	0.00 (−0.02, 0.02)	(0.35)
	25	185	0.00 (−0.2, 0.02)		0.00 (−0.02, 0.02)		179	0.00 (−0.02, 0.02)	
	101	182	0.02 (−0.01, 0.04)		0.01 (−0.01, 0.04)		176	0.02 (−0.01, 0.04)	

**Unintentional head impacts**	0	479	–	0.95 (0.62)	–	1.53 (0.47)	468	–	2.10
	1	136	0.00 (−0.02, 0.02)		−0.01 (−0.03, 0.01)		131	−0.01 (−0.03, 0.01)	(0.35)
	2+	117	−0.01 (−0.04, 0.01)		−0.01 (−0.03, 0.01)		112	−0.02 (−0.04, 0.01)	

		***N* = 705**	**Attention: higher score = better performance^f^** **One back task (ONB)**				***N* = 684**		

**Heading**	2	170	–	11.80	–	9.94 (0.02)	166	–	9.64
	9	176	0.01 (−0.03, 0.05)	(0.01)	0.01 (−0.03, 0.06)		171	−0.01 (−0.03, 0.06)	(0.02)
	25	178	−0.04 (−0.09, 0.01)		−0.04 (−0.09, 0.01)		172	−0.04 (−0.09, 0.10)	
	101	181	−0.07 (−0.12, −0.01)		−0.06 (−0.12, 0.00)		175	−0.06 (−0.12, 0.00)	

**Unintentional head impacts**	0	459	–	3.89 (0.14)	–	1.60 (0.45)	447	–	1.78
	1	133	−0.04 (−0.08, 0.01)		−0.03 (−0.07, 0.02)		128	−0.03 (−0.08, 0.02)	(0.41)
	2+	113	−0.03 (−0.08, 0.01)		−0.02 (−0.07, 0.03)		109	−0.02 (−0.07, 0.03)	

		***N* = 741**	**Working memory: higher score = better performance^g^** **Two back task (TWOB)**				***N* = 719**		

**Heading**	2	177	–	8.77 (0.03)	–	7.46 (0.06)	173	–	7.85
	9	191	0.01 (−0.03, 0.04)		0.08 (−0.03, 0.05)		186	−0.01 (−0.03, 0.05)	(0.05)
	25	187	−0.04 (−0.09, 0.01)		−0.09 (−0.16, −0.01)		181	−0.04 (−0.09, 0.01)	
	101	186	−0.07 (−0.13, −0.01)		−0.03 (−0.08, 0.02)		179	−0.07 (−0.13, −0.00)	

**Unintentional head impacts**	0	484	–	3.17 (0.21)	–	1.26 (0.53)	472	–	1.40
	1	140	−0.40 (−0.09, 0.01)		−0.03 (−0.08, 0.02)		135	−0.03 (−0.08, 0.02)	(0.50)
	2+	117	−0.03 (−0.08, 0.02)		−0.01 (−0.06, 0.04)		173	−0.01 (−0.06, 0.04)	
		
		***N* = 741**	**Verbal memory: higher score = better performance^h^** **International shopping list delayed recall (ISRL)**				***N* = 719**		
**Heading**	2	177	–	2.22 (0.53)	–	2.03 (0.57)	173	–	1.88
	9	191	0.18 (−0.18, 0.54)		0.19 (−0.17, 0.55)		186	0.18 (−0.19, 0.55)	(0.60)
	25	187	−0.06 (−0.46, 0.33)		−0.05 (−0.45, 0.35)		181	−0.06 (−0.47, 0.35)	
	101	186	−0.09 (−0.52, 0.35)		−0.07 (−0.54, 0.40)		179	−0.06 (−0.53, 0.41)	

**Unintentional head impacts**	0	484	–	0.24 (0.89)	–	0.08 (0.96)	472	–	0.16
	1	140	−0.04 (−0.37, 0.29)		−0.03 (−0.36, 0.31)		135	−0.05 (−0.40, 0.29)	(0.92)
	2+	117	−0.12 (−0.54, 0.32)		−0.06 (−0.52, 0.40)		173	−0.08 (−0.55, 0.38)	

*^a^Separate models were run for heading and for unintentional head impacts; N refers to the number of HeadCounts-2w used in the specific model 2*.

*^b^A single model was run that included both heading and unintentional head impacts*.

*^c^Covariates: gender, main position of soccer play, WRAT; and cigarette use*.

*^d^Covariates: age and WRAT*.

*^e^Covariates: gender, main position of soccer play, age and the WRAT*.

*^f^Covariates: main position of soccer play, cigarette use, and WRAT*.

*^g^Covariates: cigarette use and WRAT and unintended model added gender*.

*^h^Covariates: gender, cigarette use, WRAT, and amount of alcoholic drinks/week*.

## Discussion

Relatively little is known about the short-term effects of heading or unintentional head impacts on cognitive function, though such effects, even if transient, might inform mediation of persistent effects of repetitive brain trauma from long-term participation in soccer (9, 18, 24). While it is not known if sub-concussive head impacts contribute to persistent changes in CNS function, evidence that NP performance co-varies in relation to heading would be consistent with such a hypothesis. Previous studies have examined the relation of long-term exposure to soccer on variation in NP function in small study samples. Heading was estimated over a season, year, or lifetime (9, 18–20, 22, 23) but unintentional head impacts were not considered in these studies. Using a validated questionnaire (32) for heading activity in a 2-week period, the results of this study indicate that variation in NP function is directly related to variation in heading but not to variation in unintentional head impacts or concussion.

Our study indicates that heading activity in a 2-week period explains variation in NP function and that this relationship is not due to self-reported concussive or even probable concussive symptoms occurring in the same 2-week period. Higher levels of heading were associated with poorer performance on cognitive tasks that emphasized psychomotor speed, attention, and working memory. Notably, these domains of cognitive function are most dependent on normal structure and functioning of brain white matter tracts, the demonstrated location of pathology in concussion and also implicated, independent of concussion, in our study of soccer heading (8, 36). Moreover, significant associations with NP performance were consistently found for the upper quartile of heading activity, consistent with an exposure-response relationship. While the level of variation in NP function does not reach the level of clinical impairment, the concern raised by these findings is that long-term exposure to transient reductions in NP function from heading could translate to persistent structural changes that then lead to persistently impaired function.

Individuals with 2-week heading exposure in the fourth quartile had an average of 4.5 games and 9.0 headings/game and an average of 5.8 practice sessions and 14.2 heading/practice session. Similarly, those in the third quartile had an average of 4.0 games and 5.0 headings/game and an average of 2.1 practice sessions and 3.4 heading/practice session. The levels of activity in these two quartiles may provide insight on threshold effects.

We previously reported that CNS symptoms from head impacts were common and included mild (i.e., some pain), moderate (i.e., moderate pain, some dizziness), severe (i.e., felt dazed, stopped play, needed medical attention), and very severe (i.e., knocked unconscious) events, even though players did not report recognized concussion during the recall period (28). Notably, moderate to very severe symptoms were more strongly related to unintentional head impacts than to heading, though heading accounted for a majority of all symptoms, in part because mild symptoms were so common (73% of all symptoms). But, the occurrence of CNS symptoms, in general, may be an indicator of a head impact that is substantial enough to have a transient impact on cognitive function (37). Repeated occurrence of mild symptoms over time may also lead to persistent change in cognitive function and not allow for full recovery.

Previous studies of head injury in soccer have focused on unintentional impacts that lead to recognized concussion (30, 38–41). Person-to-person collisions, for example, have been recently reported as the dominant cause of concussion in high school players, based on sideline observer detection of overt events (42). Our previous report, however, found that such events are likely to represent a very small proportion of all moderate to severe CNS symptom events. Among 124 moderate to very severe events, 82.3% were moderate (28). Overt or covert concussions may thus not be the only or most prevalent cause of altered cognitive performance from soccer. In Model C (Table 3) removal of data with severe or very severe CNS symptoms did not influence the association of heading with NP scores.

We examined whether cognitive function varied in relation to heading activity and unintentional head impacts, not whether present clinically meaningful variation in cognitive function (i.e., clinical deficit) is related to heading. While we did not examine changes in NP function from 2-week period to 2-week period, our findings are consistent with the notion that individuals who head the ball may experience transient sub-clinical reductions in cognitive performance that we hypothesize returns to baseline performance when heading activity ceases. Notably, our prior work indicates that unintentional head impacts may be more strongly related to clinically overt CNS symptoms. However, the larger question relates to heading, which is under the control of the soccer player and is very common relative to unintentional head impacts. We do not know if there are residual effects from transient changes that may result in micro-structural changes (8, 24) and, in turn, slow the return to pre- exposure functioning. Depending on the persistence of exposure to heading, these changes may accumulate over time and contribute to sub-clinical or, eventually, overt clinical decline. Long-term follow-up of soccer players and cognitive assessment will be required to determine the relation of transient changes to long-term persistent reduction in function.

We studied relatively young adult amateur players from one area of the United States. Soccer play across the US and worldwide is diverse in the frequency and intensity of play, training, and organization. But, the range of exposure to soccer heading and unintentional head impacts is likely to be generalizable to adult amateur soccer players (43) even though we cannot specifically generalize to other groups, including adolescents and younger children.

There are several potential alternative explanations to the associations we have observed. First, the associations of heading with NP performance could be explained, in part, by a carryover effect from a previous 2-week period. In this study, soccer players completed a HeadCount-2w and NP tests every 3–6 months so we were not able to evaluate the potential impact of heading from the prior 2-week interval. In general, though, we expect that the relationship of heading to variation in NP function will be attenuated in relation to time since heading. If there is a carryover effect it reinforces the direct relation of heading and cognitive performance, as well as the persistence of the effect. However, a formal evaluation of serial heading data and actual change in NP function from one period to the next will be required to fully address this concern. Second, there may be confounding from long-term exposure to heading. While we do not have lifetime heading data, we estimated the Pearson’s *r*^2^ of 2-week heading with one-year heading to be 0.18. This relatively low correlation is not substantial enough to explain the associations we have observed. Nonetheless, we hypothesize that long-term exposure to heading can result in persistent structural changes to the CNS and persistence of impaired cognitive function, where the impact of continued heading may be more severe. Third, the heading and NP relations may be explained by unmeasured confounders. Measures of alcohol consumption, tobacco use, and head injuries outside of soccer did not influence the heading and NP relationships. It is possible that individuals who had substantial amounts of heading were anxious about their NP function and that this impaired test performance, but we would have expected such confounding to result in associations with all NP tests. Fourth, mean 2-week estimates of heading were significantly different by quartile of WRAT score. But, after adjusting for potential confounders, heading and WRAT scores were not significantly related. Nonetheless, we included WRAT score as a covariate in our models because WRAT is significantly correlated (i.e., Pearson’s *r* of 0.15) with the age that individuals started heading the ball. WRAT was not related to the GMCT scores, the cognitive test that was most strongly related to heading. WRAT was modestly related to other cognitive tests with a Pearson’s *r* ranging from 0.18 to 0.26. As such, WRAT was included as a covariate in all models.

There are several potential limitations to this study. First, the study population was comprised of volunteers. While selection bias is a potential threat to the validity of the study findings we do not believe it is a meaningful threat. The findings for heading in this study is highly consistent with what we have observed with a diversity of outcomes (symptoms, neurocognitive function, etc.) (8, 28, 32, 33) using different time scales. It is also unlikely that these findings are explained by confounding as we adjusted for the most likely external factors correlated with exposure and outcome variables. Second, we relied on self-reported exposure to heading in a 2-week period. We previously demonstrated that 2-week recall of heading is directly correlated with external observer measures of heading and, separately, with daily reporting of heading (33). Residual errors in self-reported 2-week heading exposure that were observed in our validity studies were random, not systematic. As such, errors in reporting are likely to result in an underestimate of the exposure-response relationships we have observed. Third, we recognize that the intensity and nature (e.g., rotational versus linear forces) of impacts vary substantially for both heading and unintentional head impacts. We were only able to exclude exposures that involved severe CNS symptoms. Moreover, the intensity and nature of individual headings varied within and among soccer players in ways that directly influence the difference between our observed heading measure and a quantitative measure of linear and angular accelerations. Such differences are likely to have resulted in individuals being categorized as having low heading but who actually had relatively higher quantitative exposure and individuals categorized as having moderate to high heading levels but who actually had relatively lower quantitative exposure.

Future research will benefit from more precise data on heading exposure that may offer insight on the specific qualities of heading that result in transient but sub-clinical variation NP function. Fourth, we did not collect exposure data on head impacts between testing sessions that were spaced 3 to 6 months apart as our focus was on the proximal effect of head impact exposures on variation in NP function, where exposures that are more than 2-weeks from testing were unlikely to be relevant. However, in a separate study, we examined the relation of NP function to annual versus 2-week heading exposure that indicates the NP domains that are affected do differ (10). Finally, we found that 2-week heading was significantly and inversely related to the WRAT score. However, after adjusting for age, sex, and other covariates, the relationship was not statistically significant or borderline significant. Nonetheless, we were cautious as to what this relationship might mean (e.g., a consequence of long-term exposure if 2-week and long-term exposure are correlated). We included WRAT as a covariate in the model to adjust for any residual confounding from its association with heading and performance on NP tests.

Our study reveals that variation in cognitive performance is explained by variation in heading in the previous 2-weeks but not variation in unintentional head impacts. Questions remain about the temporal relation, magnitude (e.g., frequency, intensity), and nature (i.e., linear versus rotational) of heading required to modify cognitive function, the persistence of the effect, and whether there are carryover effects mediated by long-term intensity of exposure. Answers to these questions may be important to managing risk of long-term effects from heading and underscore the need for long-term follow-up.

Current prevention efforts are focused on minimizing unintentional head impacts as these are the most common cause of *recognized concussive* injury in soccer (21). Concussive head impacts, however, may not represent the full span of risks for brain injury. Decrements in cognitive function from heading that we have observed are likely mediated by at least transient if not enduring alteration of brain function. If repeated transient changes due to heading in fact mediate long-term underlying pathophysiologic changes, then current prevention practices will be insufficient to mitigate risk of cumulative brain damage due to soccer play.

## Ethics Statement

This study was carried out in accordance with the recommendations of the Albert Einstein College of Medicine Institutional Review Board with written informed consent from all subjects. All subjects gave written informed consent in accordance with the Declaration of Helsinki. The protocol was approved by the Albert Einstein College of Medicine Institutional Review Board.

## Author Contributions

WS: study concept and design, interpretation of data, and critical revision of manuscript for intellectual content. NK, MS, MZ, MK, and RL: interpretation of data and critical revision of manuscript for intellectual content. CI: acquisition of data, analysis, and interpretation of data. ML: study concept and design, interpretation of data, critical revision of manuscript for intellectual content, and study supervision.

## Conflict of Interest Statement

The authors declare that the research was conducted in the absence of any commercial or financial relationships that could be construed as a potential conflict of interest.
